# Phylogenetic and histological variation in avipoxviruses isolated in South Africa

**DOI:** 10.1099/vir.0.054049-0

**Published:** 2013-10

**Authors:** Kristy Offerman, Olivia Carulei, Tertius A. Gous, Nicola Douglass, Anna-Lise Williamson

**Affiliations:** 1 Division of Medical Virology, Department of Clinical Laboratory Sciences, University of Cape Town, Cape Town, South Africa; 2 Specialist Veterinary Pathologist, Cape Town, South Africa; 3 Institute of Infectious Disease and Molecular Medicine, University of Cape Town and National Health Laboratory Service, Groote Schuur Hospital, Cape Town, South Africa

## Abstract

Thirteen novel avipoxviruses were isolated from birds from different regions of South Africa. These viruses could be divided into six groups, according to gross pathology and pock appearance on chick chorioallantoic membranes (CAMs). Histopathology revealed distinct differences in epidermal and mesodermal cell proliferation, as well as immune cell infiltration, caused by the different avipoxviruses, even within groups of viruses causing similar CAM gross pathology. In order to determine the genetic relationships among the viruses, several conserved poxvirus genetic regions, corresponding to vaccinia virus (VACV) A3L (*fpv167* locus, VACV P4b), G8R (*fpv126* locus, VLTF-1), H3L (*fpv140* locus, VACV H3L) and A11R–A12L (*fpv175*–*176* locus) were analysed phylogenetically. The South African avipoxvirus isolates in this study all grouped in clade A, in either subclade A2 or A3 of the genus *Avipoxvirus* and differ from the commercial fowlpox vaccines (subclade A1) in use in the South African poultry industry. Analysis of different loci resulted in different branching patterns. There was no correlation between gross morphology, histopathology, pock morphology and phylogenetic grouping. There was also no correlation between geographical distribution and virus phenotype or genotype.

## Introduction

Avipoxviruses (APVs) are large, complex DNA viruses that belong to the subfamily *Chordopoxvirinae* of the family *Poxviridae* (ICTV, 2012). They have been shown to naturally infect more than 278 of the approximately 9000 species of wild and domestic birds (Van Riper & Forrester, 2007). Despite the large number of host species, according to the International Committee on Taxonomy of Viruses, there are currently only ten defined APV species (ICTV, 2012), with species names originally assigned according to the bird species that they infect or from which they were isolated (Bolte *et al.*, 1999). As APVs are often not host specific and differ with respect to their virulence, the current means of taxonomy and classification has been criticized (Jarmin *et al.*, 2006; Manarolla *et al.*, 2010). Further characterization of this genus is therefore necessary.

Infected birds display various clinical signs of poxvirus infection, depending on the route of transmission, viral virulence and host susceptibility to the infecting strain. Cutaneous infection is characterized by nodular lesions on sparsely feathered regions of the body, and diphtheric infection usually results in higher mortality rates and produces lesions in the upper respiratory and digestive tracts of birds (Bolte *et al.*, 1999). APV infection is diagnosed by pock formation on chick chorioallantoic membranes (CAMs), histopathology (Bollinger, 1873; Eaves & Flewett, 1955), electron microscopy (Catroxo *et al.*, 2009) and/or PCR. Manarolla *et al.* (2010) described differences in gross lesions, membrane thickening and histopathology of 15 APV from northern Italy, and a recent study in Egypt described the gross pock morphologies of seven APV isolates (Abdallah & Hassanin, 2013). Case reports have also described the growth characteristics of individual APV isolates (Boosinger *et al.*, 1982; Haligur, *et al.* 2009; Kulich *et al.*, 2008; Rampin *et al.*, 2007).

APV phylogenetic studies have previously been based on the gene corresponding to vaccinia virus (VACV) P4b (*fpv*167 locus, VACV A3L) (Carulei *et al.*, 2009; Jarmin *et al.*, 2006; Lee & Lee, 1997; Manarolla *et al.*, 2010), which encodes a 75.2 kDa virion core protein, 4b, and is highly conserved among all poxviruses (Binns *et al.*, 1989). Phylogenetic analysis of this locus indicates that all strains cluster into three major clades: A [fowlpox virus (FWPV)-like], B [canarypox virus (CNPV)-like] and C (psittacine). Clades A and B can be further divided into six minor clades, namely A1, A2, A3, A4, B1 and B2 (Jarmin, *et al.* 2006). Two additional conserved genes have been used to validate the findings based on P4b: the genes encoding virion envelope protein p35 (*fpv*140, VACV H3L; Carulei *et al.*, 2009; Jarmin *et al.*, 2006; Manarolla *et al.*, 2010) and VLTF-1 (VACV G8R; *fpv*126 locus), which encodes the most conserved protein between FWPV and CNPV with 95 % amino acid identity (Carulei *et al.*, 2009; Tulman *et al.*, 2004). FWPV ORF175 and ORF176 are orthologues of conserved VACV A11R and A12L, which encode a non-structural protein involved in virion formation (Resch *et al.*, 2005) and a 25 kDa core protein involved in multiple stages of morphogenesis (Yang, 2007), respectively.

The best-characterized APVs are the species prototypes, FWPV in clade A1 and CNPV in clade B1. Both genomes have been fully sequenced and their divergence was found to be greater than that observed within other poxvirus genera (Afonso et al., 2000; Tulman *et al.*, 2004), suggesting that APVs may constitute a separate subfamily within the family *Poxviridae* (Amano *et al.*, 1999; Boyle, 2007; Tulman *et al.*, 2004).

Relatively little information is available regarding the APV strains circulating in South African birds. APV infection of an African penguin (*Spheniscus demersus*) (Carulei *et al.*, 2009; Stannard *et al.*, 1998), a flamingo (*Phoenicopterus minor*) (Zimmermann *et al.*, 2011), ostriches (*Struthio camelus australis*) (Allwright, *et al.* 1994), Cape turtle doves (*Streptopelia capicola*) (Middlemiss, 1961) and a Cape thrush (*Turdus olivaceus*) (Middlemiss, 1961) have been described. The objective of this study was to provide a more thorough investigation of APV isolates circulating in South Africa. Thirteen novel South African APVs were isolated from various birds from different locations in South Africa (Table 1). Of these samples, 11 were characterized in terms of their growth on CAMs. This was a comparative study of the macroscopic and histopathological characteristics of 11 APV isolates and is the first of its kind in sub-Saharan Africa. Phylogenetic analysis of all 13 isolates was also performed based on the three previously published loci corresponding to *fpv167* (P4b), *fpv*26 (VLTF-1) and *fpv140* (H3l) as well as an additional locus corresponding to *fpv175*–*176* (VACV A11R–A12L; Goebel *et al.*, 1990). For the first time, information is available on the APVs that are circulating in South African birds.

**Table 1.** Details of the APV isolates used in this study and summary of their characterization APVs are grouped according to their growth characteristics.

Group	Abbreviation	Host species	Symptoms	Membrane thickening	Pock morphology	Geographical source	Phylogenetic clade			
							P4b	VLTF-1	H3L	*fpv175*–*176*
1	CNPV	Canary (*Serinus canaria*)	Unknown	None	Small distinct yellow pocks	Unknown*	–	–	–	–
2	PEPV (PEPV San92)	Penguin (*Spheniscus demersus*)	Lesions around the eye	None	Pale white pocks	Cape Town, Table View**†**	A2 (FJ948105)	A2 (FJ948104)	A2 (FJ948106)	A2 (KC821590)
3	Pi2 (PGPVO Pi2)	Juvenile racing pigeon (*Columba livia domestica*)	Severe lesions	None	Large bright white pocks	Cape Town	A2 (KC821556)	A3iv (KC821568)	A3iv (KC821580)	A3iv (a) (KC821592)
4	FGPV (FGPV-KD09/ZAF)	Flamingo (*Phoenicopterus minor*)	Lesions on the legs and feet	Membrane thickening	Small pale pocks	Kimberley (Zimmermann *et al.*, 2011)	A3iii (GU204249)	A3iii (KC821561)	A3iii (KC821573)	–
5	RP2 (PGPV93K RP2)	Rock pigeon (*Columba guinea*)	Unknown	Slight thickening of CAM	Variable size white pocks	Cape Town, Claremont	A2 (KC821559)	A3iv (KC821571)	A3iv (KC821583)	A3iv (a) (KC821595)
5	LD2 (PGPV11K LD2)	Laughing dove (*Spilopelia senegalensis*)	Small diphtheric lesion in the lower beak	Slight thickening of CAM	Variable size white pocks	Port Elizabeth, Walmer‡	A3iii (KC821554)	A3i (KC821566)	A3i (KC821578)	A3i (KC821589)
5	Pi5 (PGPV11K Pi5)	Racing pigeon (*Columba livia domestica*)	Lesion around the eye	Slight thickening of CAM	Variable size white pocks	Pineview, Grabouw	A2 (KC821552)	A3iv (KC821564)	A3iv (KC821576)	A3iv (b) (KC821587)
6	FeP2 (PGPV11K FP2)	Feral pigeon (*Columba livia*)	Lesions around the eyes	Substantial thickening of CAM	Variable size white still visible	Port Elizabeth‡	A2 (KC821551)	A3iv (KC821563)	A3iv (KC821575)	A3iv (b) (KC821586)
6	LD1 (PGPV10K LD1)	Laughing dove (*Spilopelia senegalensis*)	Unknown	Substantial thickening of CAM	Variable size white still visible	Cape Town, Table View**†**	A3i (KC821553)	A3i (KC821565)	A3i (KC821577)	A3i (KC821588)
7	FeP1 (PGPV11K FP1)	Feral pigeon (*Columba livia*)	Lesions around the eyes	Severe thickening of membrane	No individual pocks visible	Port Elizabeth, Richmond Hill‡	A3ii (KC821550)	A3i (KC821562)	A3i (KC821574)	A3i (KC821585)
7	RP1 (PGPV10K RP1)	Rock pigeon (*Columba guinea*)	Unknown	Severe thickening of membrane	No individual pocks visible	Cape Town, Table View**†**	A3i (KC821558)	A3i (KC821570)	A3i (KC821582)	A3i (KC821594)
7	Pi1 (PGPVO Pi1)	Racing pigeon (*Columba livia domestica*)	Unknown	Severe thickening of membrane	No individual pocks visible	Stellenbosch§	A3i (KC821555)	A3i (KC821567)	A3i (KC821579)	A3i (KC821591)
Unassigned	Pi4 (PGPVO Pi4)	Racing pigeon (*Columba livia domestica*)	Unknown	–	–	Cape Town	A2 (KC821557)	A3iv (KC821569)	A3iv (KC821581)	A3iv (a) (KC821593)
Unassigned	SP1 (PGPV10K SP1)	Juvenile rock pigeon (speckled) (*Columba guinea*)	Severe lesions on the beak and eyes	–	–	Cape Town, Table View**†**	A3i (KC821560)	A3i (KC821572)	A3i (KC821584)	A3i (KC821596)

* From the Dumbell collection, originally from Mayr.

† From the Southern African Foundation for the Conservation of Coastal Birds.

‡ From Dr Peter Kroon: Southern Cross Veterinary Clinic.

§ From the Western Cape Department of Agriculture.

## Results

### Gross pathological and histopathological characterization of 11 South African APVs on chicken CAMs

Of the 13 novel South Africa APVs isolated, 11 were analysed in terms of their growth and histopathology on CAMs. Due to uncertain titres, Pi4 and SP1 were excluded from the histological analysis. SP1, from a speckled pigeon, caused no visible pocks on CAMs.

**Fig. 1.**
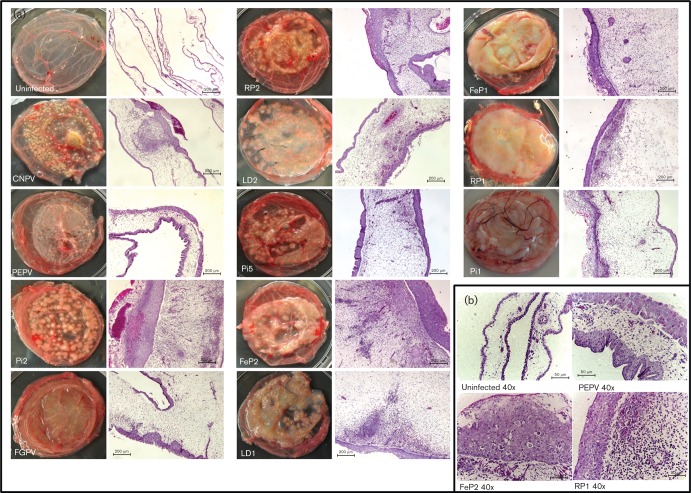
Macroscopic and histological comparison of uninfected and infected CAMs of embryonated chicken eggs. Viruses (CNPV, PEPV, Pi2, FGPV, RP2, LD2, Pi5, FeP2, LD1, FeP1, RP1, and Pi1; see Table 1 for abbreviations) (10^3^ p.f.u.) were inoculated onto the CAMs of 10–11-day-old embryonated chicken eggs. (a) Differences in pock morphology and degree of inflammation of the CAM tissue. Other observations are given in Table 2. Magnification 10×, H&E stain. Bar, 200 µm. (b) High-magnification comparison of an uninfected CAM and CAMs infected with 10^3^ p.f.u. PEPV, FeP2 and RP1 .   Magnification 40×, H&E stain. Bar, 50 µm.

The 11 APV isolates could be divided into six groups, separate from CNPV, based on pock and CAM morphology (Table 1). CNPV (isolated from a canary), penguinpox virus (PEPV; from a penguin) and Pi2 (from a racing pigeon) caused no obvious membrane thickening, but the pock lesions produced by these viruses each differed in colour, size and density (Fig. 1). CNPV infection resulted in small, yellow pocks (Fig. 1) and PEPV pocks were very small, flat and white in colour. However, the pocks resulting from Pi2 infection were large, raised, round and white with pink centres, possibly suggesting the presence of haemorrhage (Fig. 1). RP2 (from a rock pigeon), LD2 (from a laughing dove) and Pi5 (from a racing pigeon) caused slight thickening of the CAM (Fig. 1). RP2 and Pi5 presented white pocks that were variable in size, with some pocks having slightly haemorrhagic centres (Fig. 1). FeP2 (from a feral pigeon), LD1 (from a laughing dove) and flamingopox virus (FGPV; from a flamingo) displayed a substantial amount of membrane thickening (Fig. 1). The resulting pocks from FeP2 and LD1 infection were white and variable in size. FeP1 (from a feral pigeon), RP1 (from a rock pigeon) and Pi1 (from a racing pigeon) caused such extreme membrane thickening that individual pocks were not visible (Fig. 1).

The histopathology of these virally infected CAMs revealed significant differences (Fig. 1 and Table 2). Although viruses that caused severe macroscopic proliferation of the CAM were noted to have extensive mesodermal hyperplasia and less epidermal hyperplasia (see Fig. 1), a more detailed histological analysis showed all viruses to be different from one another (Table 2).

**Table 2.** A histopathological comparison of the 11 South African APVs

Virus	Macroscopic thickening of membrane	Epithelial hyperplasia		Mesodermal hyperplasia/ oedema	Angiogenesis	Fibroplasia	Inclusions	Vacuolization	Ballooning degeneration	Sloughing	Necrosis	Immune cell infiltration			Additional comments
		Chorionic epithelium	Allantoic epithelium									Lymphocytes	Heterophils	Macrophages/histocytes	
CNPV	+	++	+/+++	++	+	+++	++	++	++	++	+++	++	+	−	Focal necrotic or keratinaceous crusts; focal areas of fibroplasia and chorionic epithelial hyperplasia; mesodermal and perivascular infiltration of lymphocytes/plasma cells
PEPV	+	++	++	+	+	−	+++	+	++	+	+	+	+	−	Generalized hyperplasia of allantoic epithelium into projections, outwards away from mesoderm
Pi2	+	+++/++++	+++	+++/++++	+++	++	+++	++	+++	++	++++	+++	++++	−	
FGPV	+++	+	+	+++	++	−	−	+	++	+++	+	+	+	−	Hyperkeratosis in areas
RP2	++	+++	++	+++	+++	+	−	++	++	+++	+++	++	++	−	Granulocyte/heterophil infiltration in epidermis with necrosis
LD2	++/+++	+	+	++	+++	++	+	−	+	+	+	+++	+++	−	
Pi5	++	++	++	++	+++	+	+++	+++	+	−	−	+++	+++	−	Formation of vesicles that are not seen in others; infected cells lyse and then fuse to form a vesicle; ‘clefting’ vesicles are mostly clear with a few granulocytes and epithelial cells present; leukostasis of blood vessels
FeP2	+++	+++	++/+++	++++	+++	+++	+++	++	++	++	++	+++	+	−	Hyperplastic epithelial nests within mesodermal tissue; papilliform projections of allantoic epithelium; pale inclusions indicative of a higher lipid content; angiogenesis of surface capilliaries and leukostasis
LD1	+++	++	+	+++	+++	+/++	+	−	+++	++	+	+++	+	−	Areas of severe ballooning degeneration of epithelial cells; focal areas of heterophil and lymphocyte infiltration and fibroplasia in mesoderm; leukostasis
FeP1	++++	+++	++	++++	+	++	++	−	+	+	+	+++	++	−	Pale inclusions indicative of a higher lipid content; epithelial nests within mesodermal tissue
RP1	++++	++	+	+++	+++	+++	++	+	+	−	+	+++	+	−	Pale inclusions; beginning of vacuolization; fibroplasia and angiogenesis in mesoderm just below chorionic epithelium
Pi1	++++	++	+	++	+++	+	+	++	+++	+++	++	++	++	−	Papilliform projections of allantoic epithelium; focal severe ballooning degeneration

Histopathology was scored as: +, little; ++, moderate; +++, extensive; ++++, extreme case; /, both instances present; −, none visible.

All infected CAMs showed varying degrees of hyperplasia and hypertrophy of both epidermal and mesodermal cells. Infected tissue exhibited ballooning degeneration of keratinocytes, necrosis and large, eosinophilic intra-cytoplasmic inclusions, which are the Bollinger bodies described in poxvirus infections (Eaves & Flewett, 1955; Purcell *et al.*, 1972) (Fig. 1b). Varying degrees of heterophil and lymphocyte infiltration were most notably observed in the mesoderm and to a lesser degree in the epidermis of the infected membranes. The viruses FeP2, Pi5, LD2 and Pi2 exhibited pronounced immune infiltration, and angiogenesis was seen in the mesoderm (Fig. 1b). Hyperkeratosis and vacuolization was noted in CAMs infected with the FGPV and RP1 isolates, respectively (Table 2). Hyperplastic epithelial nests were noted in the mesoderm of FeP2 and FeP1 (Table 2). Angiogenesis and fibroplasia were observed to varying degrees in most isolates (Table 2).

### Phylogenetic analysis of APVs in South Africa

Nucleotide and amino acid sequences corresponding to VACV *fpv26* (VLTF-1), *fpv167* (P4b), *fpv140* (H3L) and *fpv175*–*176* (VACV A11R–A12L) were aligned with published sequences obtained from GenBank, and phylogenetic relationships were determined based on these alignments. Because of the highly conserved nature of the genes analysed, nucleotide sequences rather than amino acid sequences were used to determine divergence (Carulei *et al.*, 2009; Jarmin *et al.*, 2006). Clades and subclades have been named according to previous APV phylogenetic studies based on the P4b gene locus (Gyuranecz *et al.*, 2013; Jarmin *et al.*, 2006).

### P4b (VACV A3L, *fpv167* locus)

The P4b gene was amplified by PCR and gave the expected 578 bp product for all 13 of the virus isolates (data not shown). A maximum-likelihood (ML) tree was constructed using the Tamura three-parameter model with gamma distribution (Tamura, 1992), with a bootstrap test of 100 replicate samples.

**Fig. 2.**
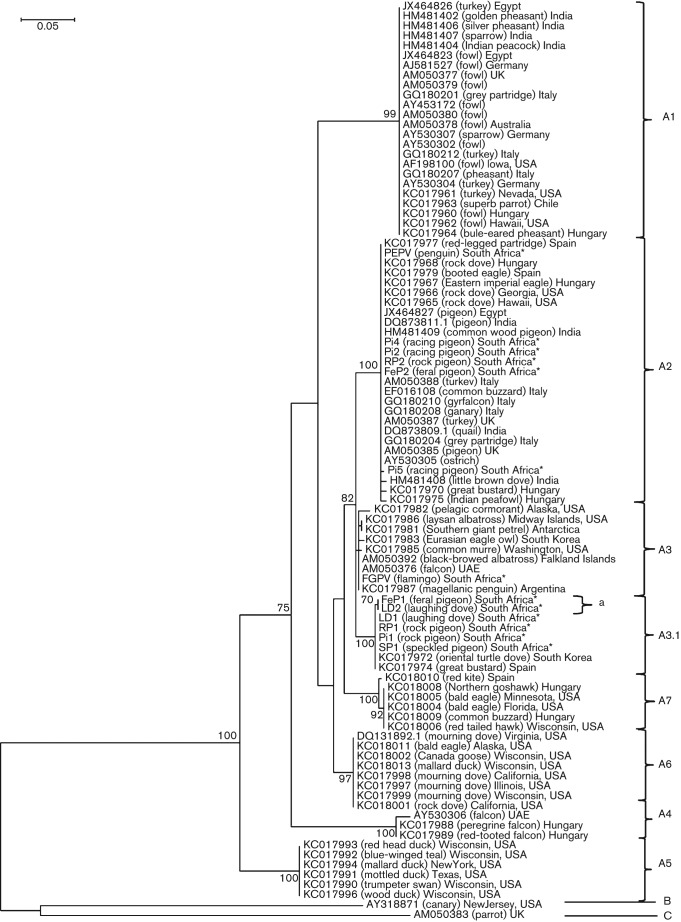
ML tree based on the muscle nucleotide alignments of P4b (*fpv167*, VACV A3L). South African isolates (CNPV, PEPV, Pi2, FGPV, RP2, LD2, Pi5, FeP2, LD1, FeP1, RP1 and Pi1) (indicated with asterisks; see Table 1 for abbreviations) were aligned with published sequences from GenBank. The tree was constructed using the Tamura three-parameter model with gamma distribution and a bootstrap test of 100 replicate samples. Entries are given as GenBank accession number, host and country of origin. Bar, nucleotide substitutions per site.

The ML tree based on nucleotide sequences at this locus (Fig. 2) clearly distinguished between known APV clades and subclades. All 13 isolates analysed in this study grouped in clade A (FWPV-like viruses) with strong bootstrap support (Fig. 2). The isolates PEPV (Carulei *et al.*, 2009), Pi4, Pi2, RP2 and FeP2 grouped in subclade A2 and shared 100 % nucleotide identity with the rest of the subclade (Fig. 2). Pi5 had a single nucleotide mutation and branched off from this subclade (Fig. 2). SP1, Pi1, RP1 and LD1 shared 100 % nucleotide identity, grouping together with an isolate from a South Korean oriental turtle dove (Gyuranecz *et al.*, 2013) and a Spanish great bustard (Gyuranecz *et al.*, 2013), in a new branch of subclade A3, annotated here as subclade A3.1 (Fig. 2). FeP1 and LD2 both exhibited one synonymous mutation in these sequences and grouped in subclade A3.1a (Fig. 2). FGPV was placed in the original subclade A3, as annotated by Jarmin *et al.* (2006), and was most closely related to isolates from a black-browed albatross (*Thalassarche melanophrys*, from the Falkland Islands, UK), a laysan albatross (*Phoebastria immutabilis*, from Midway Islands, USA), a pelagic cormorant (*Phalacrocorax pelagius*, from Alaska, USA), a southern giant petrel (*Macronectes giganteus*, from Antarctica), a Eurasian eagle owl (*Bubo bubo*, from South Korea), a common murre (*Uria aalge*, from Washington, USA), a falcon (*Falco* sp., from United Arab Emirates) and a magellanic penguin (*Speniscus magellanicus*, from Argentinia) (Fig. 2).

### VLTF-1 (VACV G8R, *fpv126* locus)

**Fig. 3.**
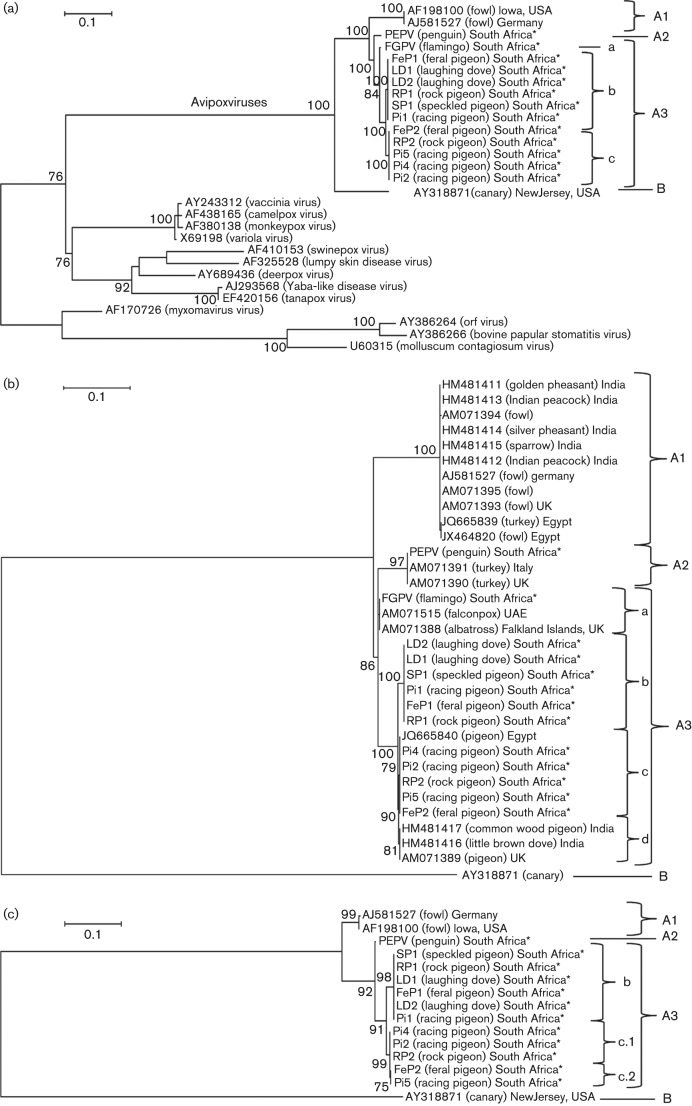
ML trees based on the muscle nucleotide alignments of the regions corresponding to VLTF-1 (VACV G8R; *fpv126* locus) (a), p35 (*fpv140*, VACVL H3L) (b) and *fpv175*–*176* (VACV A11R–A12L) (c). The South African isolates (CNPV, PEPV, Pi2, FGPV, RP2, LD2, Pi5, FeP2, LD1, FeP1, RP1 and Pi1) (indicated with asterisks; see Table 1 for abbreviations) were aligned with published sequences from GenBank. ML trees were constructed using the Tamura three-parameter model with gamma distribution, with a bootstrap test of 100 replicate samples. Entries are given as GenBank accession number, host and country of origin (a–c), or GenBank accession number and disease (a). Bar, nucleotide substitutions per site.

All 13 South Africa APV isolates produced the expected 700 bp product upon PCR amplification. These products were sequenced in duplicate and truncated to 570 bp for alignment with published VLTF-1 orthologues. A ML tree was constructed using the Tamura three-parameter model with gamma distribution and the rate variation model allowed for some sites to be evolutionarily invariable [(+*I*), 28.7650 % sites]. The ML tree based on the VLTF-1 nucleotide sequence alignment (Fig. 3a) showed that the South Africa isolates belonged to the genus *Avipoxviruses* and grouped with FWPV, in a separate clade from CNPV. Additionally, VLTF-1 provided greater resolution of clade A viruses. PEPV grouped alone in subclade A2; FeP1, LD1, LD2, RP1, SP1 and Pi1 grouped together within subclade A3 (A3b) with 100 % nucleotide identity; FeP2, RP2, Pi5, Pi4 and Pi2 also grouped together with 100 % nucleotide identity within subclade A3 (A3c); and FGPV grouped separately from these two groups of columbiforme isolates in subclade A3a.

### H3L (VACV H3L, *fpv140* locus)

Amplification of this region produced positive results of 1100 bp for all 13 viruses (data not shown). Upon sequencing, these products were trimmed to 718 bp and aligned with the available published APV sequences at this locus. An ML tree was constructed using the Tamura three-parameter model with gamma distribution.

The ML tree based on the nucleotide sequence of H3L (*fpv140* locus) (Fig. 3b) also grouped Pi4, Pi2, RP2, Pi5 and FeP2 in subclade A3 (A3c). According to phylogenetic analysis of P4b, these viruses grouped in subclade A2. These viruses were most closely related to pigeonpox virus Peekham (PGPVP, GenBank accession no. AM071389), isolated in the UK (Jarmin *et al.*, 2006), with 99.72 % nucleotide identity. The viruses RP1, FeP1, Pi1, SP1, LD1 and LD2 also grouped in subclade A3 (A3b) and shared 98.75 % nucleotide identity with Pi4, Pi2, RP2, Pi5 and FeP2.

### *fpv175*–*176* (VACV A11R–A12R)

Amplification of this region produced the expected 700 bp product for all isolates except FGPV, which did not give a product (data not shown). An ML tree was constructed using the Tamura three-parameter model with gamma distribution. The tree based on the nucleotide sequences of this conserved region (Fig. 3c) provided even further resolution of subclade A3c, grouping the viruses Pi5 and FeP2 (A3c.2) separately from RP2, Pi4 and Pi2 (A3c.1) with strong bootstrap support. At the other two loci, these three viruses shared 100 % nucleotide identity, except for the P4b gene, where Pi5 had a single base pair difference.

## Discussion

This study compared the gross pathological and histopathological characteristics of CAMs following infection by 11 APVs isolated from different bird species from diverse regions of South Africa. Poxvirus growth on CAMs generally produces raised, circular lesions, or ‘pocks’, of varying morphology. Studies describing the gross pathology and histology of different APVs in CAMs have been carried out elsewhere, including Italy and Egypt. This is the first comparison of the growth characteristics of different APVs isolated from various bird species in South Africa. Different APVs were grown using the same method, and each virus stock was titrated so that a constant amount of virus was inoculated onto each CAM. This allowed accurate comparisons of growth characteristics among viruses isolated from different bird species and geographical regions.

Manarolla *et al.* (2010) reported variable levels of thickening, ranging from mild to severe, in CAMs infected with APV isolates from Italy (Manarolla *et al.*, 2010). In an Egyptian study, isolates from chickens and a turkey produced compact, greyish-white pocks and marked thickening of the infected CAM tissue (Abdallah & Hassanin, 2013). In this same study, a pigeon poxvirus (PGPV) isolate produced nodular yellowish pocks and moderate thickening of the CAM tissue (Abdallah & Hassanin, 2013). South Africa APV isolates also exhibited differing pock morphologies and degrees of membrane thickening (Tables 1 and 2). Interestingly, all viruses isolated from pigeons (Pi2, RP2, Pi5 and FeP2) produced white pocks of variable size except for those isolates where the membrane thickening was so severe that no individual pocks were visible (FeP1, RP1 and Pi1). At lower titres (10^2^ and 10^1^) where membrane thickening was reduced, these viruses produced distinct white pocks (not shown). This pock morphology in South African PGPV isolates was different from the yellowish nodular pocks seen in CAMs infected with an Egyptian PGPV isolate (Abdallah & Hassanin, 2013).

There have been many reports that describe differences in growth characteristics of the orthopoxviruses (Archard & Mackett, 1979; Archard *et al.*, 1984; Bedson & Dumbell, 1961; Martinez-Pomares *et al.*, 1993; Roth *et al.*, 2012). Factors that influence poxvirus growth on CAMs include incubation temperature (Bedson & Dumbell, 1961), age of embryos and the source of eggs (Baxby, 1969). Variability in pock colour has also been ascribed to mutation of specific viral genes (Archard & Mackett, 1979; Archard *et al.*, 1984). Unlike the pock phenotype of most other orthopoxviruses, wild-type cowpox virus (CPV) produces haemorrhagic red pocks on CAMs. However, CPV can produce spontaneous white-pock variants resulting from the deletion or mutation of a specific gene encoding the cytokine response modifier A (CrmA; SPI-2) protein (Archard & Mackett, 1979; Archard *et al.*, 1984). On histological examination, the CPV red pock is shown to lack inflammatory cells and have increased virus antigen and infectivity levels (Palumbo *et al.*, 1989). The CPV white-pock phenotype is characterized by the presence of large numbers of heterophils and macrophages (Palumbo *et al.*, 1989; Roth *et al.*, 2012) and produces extensive thickening of CAM tissue caused by proliferation of the epidermal and mesodermal cells (Chua *et al.*, 1990). Therefore, different phenotypes or growth characteristics may be indicative of different levels of immune response in the CAM tissue (Palumbo *et al.*, 1989; Roth *et al.*, 2012), caused by the genetic make-up of the virus.

The pathologies of all the virus-infected CAM tissues in this study, including thickening of the membrane and immune cell infiltration, are suggestive of an acute inflammatory response. The chicken embryo at 10–15 days old lacks a functional specific immune system (Eerola *et al.*, 1987; Dibner *et al.*, 1998) and therefore the CAM model can be used to analyse virus-induced host responses in the absence of specific adaptive immune responses (Fredrickson *et al.*, 1992; Palumbo *et al.*, 1994). The morphological and histological differences observed among APVs in this study (Fig. 1, Tables 1 and 2) could be attributed to the absence or presence of specific immunomodulatory gene products, which may influence inflammation. As the viruses in this study were grown using the same protocol on eggs from the same source, one can assume that the variation in pock and CAM presentation is due to differences in genetic content of the respective viruses.

Several specific genes have been associated with differences in phenotype of different poxviruses. Genes encoding serine proteinase inhibitors (serpins), such as CPV CrmA (SPI-2, B13R; Turner *et al.*, 1999), are found in most chordopoxviruses; for example, VACV B13R, myxoma virus Serp2 and ectromelia virus SPI-2 are all homologues of CPV CrmA. VACV C22L encodes a TNF receptor homologue, which inhibits inflammation (Palumbo *et al.*, 1994; Rathinam *et al.*, 2012). FWPV encodes five serpin homologues (*fpv010*, *fpv040*, *fpv044*, *fpv204* and *fpv251*; Afonso *et al.*, 2000) and two homologues of cellular β-nerve growth factor (β-NGF) (*fpv*072, *fpv*076), which, when expressed by the virus, may interfere with early innate immune responses and may be important for viral infection (Afonso *et al.*, 2000). FWPV also encodes a gene similar to IL-18-binding protein (*fpv073*), which may inhibit inflammation (Afonso *et al.*, 2000). It is possible that the viruses that do not cause significant inflammation, such as PEPV (penguin), CNPV (canary), Pi2 and Pi5 (racing pigeon), LD2 (laughing dove) and RP2 (rock pigeon), may contain one or more of these anti-inflammatory genes or novel anti-inflammatory genes. These genes may be responsible for their phenotype on CAMs. Whole-genome sequencing and gene function analysis will be necessary to determine the cause of the different growth phenotypes of these viruses.

Differences in virus-induced responses in the CAMs, such as membrane thickening, immune cell infiltration, angiogenesis and hyperplasia, were observed in this study, and one can only speculate why these differences exist. In the CAM model, administration of transforming growth factor β1 (TGF-β1) initiates a response that is similar in appearance to the CAM tissue infected by the isolates that caused extensive inflammation, namely Pi1 (racing pigeon), FeP1 (feral pigeon), RP1 (rock pigeon), FeP2 (feral pigeon), LD1 (laughing dove) and FGPV (flamingo) (Yang & Moses, 1990). TGF-β1 has pro-inflammatory properties and can inhibit growth, increase cellular accumulation through chemotaxis or cellular migration, and increase microvascular angiogenesis. It is important in wound healing, tumour progression and embryogenesis (Durum & Oppenheim, 1993; Yang & Moses, 1990). The isolates mentioned above caused epithelial and mesodermal thickening due to cellular hypertrophy and hyperplasia, angiogenesis, sloughing and infiltration of mononuclear immune cells, which was similar to the appearance of CAM tissue that has been treated with TGF-β1 (Yang & Moses, 1990). FWPV (*fpv080*) encodes a homologue of the eukaryotic TGF-β, which is thought to be involved in suppression of the host immune response and/or cell growth and differentiation (Afonso *et al.*, 2000). It is possible that the viruses that cause inflammation (FeP1, RP1 and Pi1) could encode functional homologues of a TGF-like gene. The proliferative diseases caused by several poxviruses, including molluscum contagiosum virus have been attributed to the production of epidermal-like growth factors (EGF-like) by virus-infected cells (Brown *et al.*, 1985; Postlethwaite, 1970). Poxvirus EGF-like growth factors have been shown to stimulate cell proliferation at regions of virus replication (McFadden *et al.*, 1996). FWPV (*fpv211*) also encodes an EGF-like domain (Afonso *et al.*, 2000) and may contribute to the hyperplasia observed in FWPV-infected tissue (Tripathy, 1991). FWPV (or PEPV) does not produce extensive membrane thickening; however, a degree of hyperplasia is observed when compared with uninfected CAM tissue. The viruses causing inflammation (Pi1, FeP1, RP1, FeP2, LD1 and FGPV) may contain additional growth factor-like genes, which may cause the increased inflammation observed in CAMs infected with these viruses.

Although the variation in pock morphology and histology among these viruses indicated that many of our novel APVs differed significantly, the phylogenetic analysis of four conserved regions suggested that these viruses are closely related to one another. For example, phenotypically, Pi5 (racing pigeon) and FeP2 (feral pigeon) differ considerably, with FeP2 causing more hyperplasia and membrane thickening than Pi5. Phylogenetically, however, they grouped together in subclade A3c.2 (according to the VLTF-1, H3L and *fpv175*–*176* loci). In addition, the isolates LD2 (laughing dove) and FeP1 (feral pigeon), which were both obtained from the same geographical region (Port Elizabeth) differed with regard to their pock morphology and histology but clustered together in the subclade A3b. Sequencing of a few conserved loci is therefore not sufficient to differentiate viruses that could be significantly different from one another. More detailed analyses, in the form of genomic sequencing, pathway analysis/immunomodulation by microarray, will help to explain why these differences exist.

It is important to note that the viruses in this study were isolated from discrete geographical locations, up to nearly 1000 km apart (Cape Town to Kimberly, 975 km; Cape Town to Port Elizabeth, 790 km; Kimberly to Port Elizabeth, 743 km). This geographical separation did not, however, coincide with clustering of the viruses according to the trees, with isolates from the same region grouping separately. Although FeP1 and LD2, both from Port Elizabeth, grouped together in subclade A3b, FeP2, from the same region in Port Elizabeth, grouped in subclade A3c (according to the ML trees constructed based on the H3L, VLTF-1 and *fpv175*–*176* loci). All three viruses from Port Elizabeth differed with respect to CAM morphology. Moreover, several viruses from different regions clustered together phylogenetically. This was seen in RP1, isolated from a rock pigeon in Table View, Cape Town, which clustered together in subclade A3b with FeP1 (feral pigeon) and LD1 (laughing dove) isolated in Port Elizabeth. Pi5 (racing pigeon) and FeP2 (feral pigeon) also clustered together in subclade A3c, and were isolated from Grabouw in the Western Cape, and from Port Elizabeth, respectively. These A3b and A3c viruses differed with respect to pock and CAM morphology.

In a similar study conducted in New Zealand, where APV infection is known to be endemic in free-ranging bird populations, it was shown that most New Zealand avipoxvirus isolates, including those isolated from a song thrush (*Turdus philomelos*), saddlebacks (*Philesturnus carunculatus rufusater*, *Philesturnus carunculatus carunculatus*), sparrow (*Passer domesticus*), black robin (*Petroica traversi*), silvereye (*Zosterops lateralis*), shore plovers (*Thinornis novaeseelandiae*), variable oyster catchers (*Haematopus unicolor*) and a paradise shelduck (*Tadorna variegate*), belonged to subclade A1, sharing 100 % nucleotide identity with the FWPV vaccine strain used in New Zealand (Ha *et al.*, 2011). This suggests that several New Zealand free-ranging birds are susceptible to the specific A1 strain used as an attenuated fowlpox vaccine. Certain New Zealand samples grouped in subclades A3 and B1 (Ha *et al.*, 2011). APVs isolated from South African birds all grouped within clade A (FWPV-like viruses), in either subclade A2 or A3. Although we know that FWPV exists in South African poultry, none of the viruses analysed in our study shared similarity to the FWPV or FWPV vaccine strains used in South Africa (clade A1) (data not shown).

Based on the phylogenetic analysis of four conserved regions, the viruses characterized from South African columbiformes cluster into two groups. The viruses from feral pigeon (FeP2), rock pigeon (RP2) and racing pigeon (Pi5) grouped in subclade A3c and the viruses from a rock pigeon (RP1), two from laughing doves (LD1 and LD2), a feral pigeon (FeP1), and a juvenile rock pigeon (SP1) grouped in subclade A3b. Therefore, in this study as well as others (Ha *et al.*, 2011; Jarmin *et al.*, 2006; Manarolla *et al.*, 2010), APVs from the same species of bird are classified in different subclades. Conversely, it has also been shown that the same viruses can infect different birds (Abdallah & Hassanin, 2013; Adams *et al.*, 2005; Pawar *et al.* 2011). Pigeonpox viruses (PGPVTP2, PGPVP, HM481409 and HM481408) group in subclade A2 according to P4b (Jarmin *et al.*, 2006; Lüschow *et al.*, 2004; Pawar *et al.*, 2011), and based on the H3L gene, they group in subclade A3, along with isolates from an albatross, falcon and flamingo (Abdallah & Hassanin, 2013; Jarmin *et al.*, 2006; Pawar *et al.*, 2011). Pigeonpox isolates grouping in subclades B1 and B2 (Jarmin *et al.*, 2006; Manarolla *et al.*, 2010; Weli *et al.*, 2004) have also been noted.

The complicated nature of the host range of APVs has led to the suggestion that the taxonomy of these viruses should be changed. Jarmin *et al.* (2006) criticized the host species-based approach to APV taxonomy because sequences taken from a particular species can be found in different subclades or clades (Jarmin *et al.*, 2006). This was seen in our study where isolates from feral pigeons, FeP1 and FeP2, grouped separately (subclade A3b and A3c, respectively) and also differed considerably with regard to their growth characteristics. Similarly, this was also seen in viruses isolated from two rock pigeons, RP1 and RP2. Therefore, the results of this study, along with several others (Abdallah & Hassanin, 2013; Jarmin *et al.*, 2006; Manarolla *et al.*, 2010; Pawar *et al.*, 2011), provide evidence that the existing host species-based classification may be oversimplified for the complicated host range of APVs.

Preliminary phylogenetic analysis and characterization of the pathology of novel South African APVs on CAMs was performed in this study. For the first time, information is available on which APVs are circulating in South African birds. According to the phylogenetic analyses presented here, the viruses circulating in South African birds group with FWPV-like viruses in clade A, subclades A2 and A3, and are shown to cluster into two groups, which are seemingly independent of the species of bird from which they were isolated. Current convention is to name the virus after the species in which it was originally described; however, it is suggested that alterations to the existing taxonomy of APV be made that take into account genetic diversity and the variability of virus–host interactions, growth characteristics and infectivity. Thus far, the genomes of only three APVs have been published; a pathogenic US strain of fowlpox (FPVUS; Afonso *et al.*, 2000), a plaque-purified, tissue-culture-adapted, attenuated European strain of FWPV (FP9; Laidlaw & Skinner, 2004) and a virulent CNPV (CNPVATCC VR-111) isolate (Tulman *et al.*, 2004). FPVUS and FP9 group in clade A1 and CNPV ATTC VR-111 groups in clade B1. According to the genetic regions *fpv26* (VLTF-1), *fpv167* (P4b), *fpv140* (H3l) and *fpv175*–*176* (VACV A11R–A12L), the novel APVs analysed in this study are grouped differently from the strains whose genome sequences have been published (Afonso *et al.*, 2000; Laidlaw & Skinner, 2004; Tulman *et al.*, 2004). More detailed analyses, in the form of genomic sequencing as well as pathway analysis/immunomodulation by microarray will allow a more thorough differentiation of APVs.

## Methods

### 

#### Virus isolates.

Lesions from infected birds were obtained from several sources throughout South Africa (Table 1). Small sections (~2 mm) of the samples were homogenized in McIllvains buffer (4 mM citric acid, 0.2 M Na_2_HPO_4_.12H_2_O, pH 7.4) using a Tenbrook grinder and centrifuged at 14 000 r.p.m. (Eppendorf Centrifuge 5417C) for 5 min. The supernatants, containing virus, were collected and used for growth in eggs. In total, 13 APV samples were isolated from six different bird species (Table 1).

#### Virus growth and titration.

Virus isolates were grown and titrated on CAMs of embryonated 10–11-day-old chicken eggs using a method described by Joklik (1962), Stannard *et al.* (1998) and Kotwal & Abrahams (2004) in order to produce high-titre viral stocks for further characterization. Each virus was grown on two species of eggs, and the gross pathology of virally infected membranes was the same. Specific-pathogen-free White Leghorn eggs were obtained from Avifarms Ltd and healthy Cobb Avian 48 eggs were obtained from a commercial company; the health status of the layers was checked by an experienced veterinarian. It has been shown that there is no difference between the growth characteristics of virus grown in commercial and specific-pathogen-free eggs (Manarolla *et al.*, 2010). To titrate virus stocks, serial dilutions were made of each stock in PBS containing penicillin (500 U ml^−1^), streptomycin (100 µg ml^−1^) and Fungin (1 µg ml^−1^), inoculated onto CAMs in triplicate as above and incubated at 37 °C for 4 days post-infection. Thereafter, the membranes were spread out on Petri dishes and the mean number of pocks per dilution was determined. The p.f.u. ml^−1^ was determined by the following equation: mean number of pocks×dilution factor×10.

#### Histopathology.

For histopathological analysis, 10-day-old commercial Cobb Avian 48 chick CAMs were inoculated with 10^3^ p.f.u. each virus, and incubated for 5 days at 37 °C. This titre was chosen for analysis as it gave a good indication of virus growth differences on CAMs. Higher titres were seen to be pathogenic to the chicks, and lower titres did not produce confluent membranes. Gross pathology was determined many times on different batches of eggs, and the growth characteristics of the respective viruses did not differ. Three eggs were inoculated for each isolate, and a representative membrane was chosen. Thereafter, virally infected CAMs were photographed, harvested and fixed in 10 % buffered formalin [formaldehyde (37–40 %), NaH_2_PO_4_.H_2_0 (35.03 M), Na_2_HPO_4_ (anhydrous, 21.84 M), made up to 1 l with distilled water; pH 7.4]. Infected portions of tissue with similar pock densities were chosen and cut for histopathology. These were rolled up, including multiple pocks in each, embedded in paraffin, cut into 4 µm sections and stained with conventional haematoxylin and eosin. Slides were examined and photographed under a light microscope.

#### PCR amplification and sequence analysis.

Viral DNA was extracted using the following method. Proteinase K was added to the virus preparation at 2 mg ml^−1^ and incubated at 55 °C for 30 mins. Thereafter, an equal volume of lysis buffer containing 10 % *N*-lauryl sarcosinate, 50 mM Tris/HCl (pH 7.8) and 200 mM β-mercaptoethanol was added before further incubation at 55 °C overnight. An equal volume of phenol : chloroform (1 : 1) was added before inversion and centrifugation at 14000 r.p.m. (Eppendorf Centrifuge 5417C) for 5 mins. RNase (100 µg ml^−1^) was added and incubated at 37C for 1 h, and conventional phenol : chloroform extraction with sodium acetate and ethanol precipitation was then performed.

PCR was performed using previously described primers for the P4b and H3l loci (Jarmin *et al.*, 2006). For VLTF-1 and *fpv175*–176 (VACV A11R–A12L), the following primers were used to amplify 700 bp products for both regions: VLTF-1 forward primer: 5′-TAAATGAGTTTGCGTATAAAAATCGATAAG-3′, and VLTF-1 reverse primer: 5′-TTCAGCATCCATAACTATCTTTGACTC-3′; *fpv175*–*176* forward primer: 5′-GGTACCGTATATTTCTATAAAACAATATCAC-3′, and *fpv175*–*176* reverse primer: 5′-ACTAGTGCTAAATCATATTAATGCTATTACGG-3′.

A 2× PCR mix (Immomix; Bioline) was used according to the manufacturer’s instructions, and PCR thermocycling was performed in a GeneAmp PCR system (Applied Biosystems).

Amplicons were purified using a commercial kit (DNA Clean and Concentrator-25; Zymo Research), and sequenced using a BigDye Teminator v3.1 sequencing kit (Applied Biosystems) using an ABI3130xl sequencer (Applied Biosystems) by the University of Stellenbosch Central Analytical Facility.

Sequence analysis was performed using CLC Bio Main Workbench software and mega5 (Tamura *et al.*, 2011). Appropriate models for each dataset were tested using mega5 and ML trees were constructed based on muscle nucleotide alignments of the sequences of P4b, VLTF-1, H3l and *fpv175*–*176*, each with a bootstrap test of 100 replicate samples.
